# *In vivo *analysis of *Caenorhabditis elegans *noncoding RNA promoter motifs

**DOI:** 10.1186/1471-2199-9-71

**Published:** 2008-08-05

**Authors:** Tiantian Li, Housheng He, Yunfei Wang, Haixia Zheng, Geir Skogerbø, Runsheng Chen

**Affiliations:** 1 Bioinformatics Laboratory and National Laboratory of Biomacromolecules, Institute of Biophysics, Chinese Academy of Sciences Beijing 100101, PR China; 2 Bioinformatics Research Group, Key Laboratory of Intelligent Information Processing, Institute of Computing Technology, Chinese Academy of Science, Beijing 100080, PR China; 3 Chinese National Human Genome Center, Beijing 100176, PR China; 4 Graduate School of the Chinese Academy of Science, Beijing 100080, PR China; 5 Shanxi Agricultural University, Taigu, Shanxi, 030801, PR China

## Abstract

**Background:**

Noncoding RNAs (ncRNAs) play important roles in a variety of cellular processes. Characterizing the transcriptional activity of ncRNA promoters is therefore a critical step toward understanding the complex cellular roles of ncRNAs.

**Results:**

Here we present an *in vivo *transcriptional analysis of three *C. elegans *ncRNA upstream motifs (UM1-3). Transcriptional activity of all three motifs has been demonstrated, and mutational analysis revealed differential contributions of different parts of each motif. We showed that upstream motif 1 (UM1) can drive the expression of green fluorescent protein (GFP), and utilized this for detailed analysis of temporal and spatial expression patterns of 5 SL2 RNAs. Upstream motifs 2 and 3 do not drive GFP expression, and termination at consecutive T runs suggests transcription by RNA polymerase III. The UM2 sequence resembles the tRNA promoter, and is actually embedded within its own short-lived, primary transcript. This is a structure which is also found at a few plant and yeast loci, and may indicate an evolutionarily very old dicistronic transcription pattern in which a tRNA serves as a promoter for an adjacent snoRNA.

**Conclusion:**

The study has demonstrated that the three upstream motifs UM1-3 have promoter activity. The UM1 sequence can drive expression of GFP, which allows for the use of UM1::GFP fusion constructs to study temporal-spatial expression patterns of UM1 ncRNA loci. The UM1 loci appear to act in concert with other upstream sequences, whereas the transcriptional activities of the UM2 and UM3 are confined to the motifs themselves.

## Background

Genome wide analyses have in recent years revealed an increasing number of noncoding RNAs (ncRNAs) [1-12], however, the functional roles of these ncRNAs are mostly still unknown. Characterizing the transcriptional activity of the promoters of these loci could be a useful step towards revealing their functional roles. Extensive analysis of ncRNA promoters have been carried out in human, *Drosophila *and yeast [13-18]. There are more than 200 known short ncRNAs loci reported in *C. elegans *(microRNAs and tRNAs not included) and a recent tiling microarray study suggests the existence of an additional 1200 short transcripts with unknown function (TUFs) [1]. However, compared to the extensive work on promoter of protein coding genes, few analyses of ncRNA promoter activity have been performed in this species [19-24].

Analysis of the 100 bp upstream sequences of 161 *C. elegans *ncRNAs using the MEME software [25] detected three distinct 50 bp upstream motifs (upstream motifs 1~3, henceforth UM1-3) [6]. Among the 161 ncRNAs, UM1 is found at the loci of 54 ncRNAs, including 23 snRNAs, 11 snoRNAs and 11 snlRNAs. UM2 is found at the loci of 47 ncRNAs, of which 40 are snoRNAs. UM3 is found at the loci of 9 ncRNAs, all are stem-bulge RNAs (sbRNAs). Of the 1222 transcripts of unknown function (TUF)[1], UM1-3 are found at 76, 44 and 4 loci, respectively.

**Figure 1:**
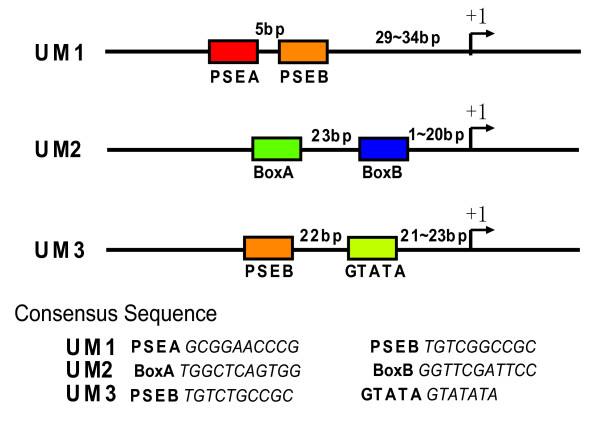
**Structures of the three putative promoters of *C. elegans *noncoding RNAs.** The most invariant submotifs in each promoter have been indicated by boxes. "+1" corresponds to the first base of the mature endogenous RNA sequence.

The core sequence of the 50 bp long UM1 is the 21 bp long snRNA proximal sequence element (PSE) [26]. This core sequence is composed of two sub-motifs spaced by 5 bp (Figure 1). In the *Drosophila *PSE the corresponding sub-motifs have been denoted as PSEA and PSEB [27] and we have used the same denotation here. Most of the *C. elegans *PSE/UM1 loci are TATA-less, and transcripts generated from such loci generally carry a 5'-end cap, suggesting transcription by polymerase II [6].

In the second motif (UM2) the most invariant sub-motifs are spaced by 33 bp and strongly resemble the Box A and B motifs of the tRNA internal promoter (Figure 1), which is known to bind transcription factor IIIC (TFIIIC) [28]. It is possible that UM2 is derived from tRNA genes that have served as promoters for downstream ncRNA genes, as similar tRNA-snoRNA dicistronic transcriptional structures have been described in plants and yeast [29-32]. Most of the UM2 loci encode snoRNAs which generally produce uncapped transcripts terminated at an oligo-T tract, and are thus likely to be transcribed by polymerase III [6], though the lack of a cap could also be due to processing of the primary snoRNA transcript [32,33].

The third motif (UM3) resembles the PSE/UM1 in that they both contain the PSEB sub-motif, but UM3 lacks PSEA, and has in addition a structure with the consensus motif GTATA located closer to the ncRNA transcription start site (TSS; Figure 1). UM3 is exclusively found at stem-bulge RNA (sbRNA) loci. The sbRNAs are terminated at an oligo-T tract, and most appear to be uncapped, indicating transcription by RNA polymerase III [6].

Short ncRNA loci in *C. elegans *are frequently found in introns of protein coding genes [2,6]. Such loci may or may not have an upstream motif. Previous analyses have found that for ncRNAs located in introns, the expression levels of intronic ncRNA loci not containing any obvious upstream motifs are closely correlated to the expression levels of the host genes [34]. On the other hand, for intronic ncRNA locus containing an upstream motif, the expression of the ncRNA locus is uncorrelated to the host gene expression, indicating that ncRNA loci with upstream motifs are independently transcribed [34].

No specific analysis of the ncRNA promoters appears to have been carried out in *C. elegans*, however, previous analysis has shown that a fragment including the first 162 bp upstream of the SL4 RNA (a variant of SL2 RNA, CeN7, which locus contains PSE/UM1) is sufficient to drive *LacZ *expression [35], and mutation of four bases in the PSEB submotif resulted in a 10-fold reduction in transcription of an SL2 RNA [36]. We here demonstrate the transcriptional activity of the three *C. elegans *ncRNA promoters. The roles of the most invariant sub-motifs were investigated by mutation analysis, and the extent of upstream sequence with influence on the ncRNA transcriptional activity was analysed.

## Results

In order to analyse the transcriptional activities of the three common ncRNA motifs, we made constructs containing varying amounts (~100 bp, ~300 bp and ~1 kb) of upstream sequences (including 30–70 bp of transcribed sequence from each selected ncRNA locus) fused to the green fluorescent protein (GFP) open reading frame (ORF). Constructs containing approximately 100 bp upstream sequences (denoted by LOCUS NAME_100) were used to test the inherent transcriptional activity of each upstream motif, whereas constructs with longer upstream sequences (LOCUS NAME_300, LOCUS NAME_1kb) were employed to investigate the possibility of additional regulatory elements.

### All the three upstream motifs are transcriptionally active

**Figure 2:**
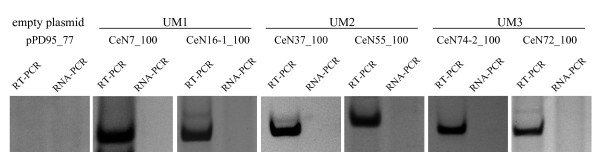
**Upstream motifs drive reporter gene expression.** Expression of the reporter gene was detected by RT-PCR using DNase I digested RNA extracted from transgenic worms as template. For the negative controls (RNA-PCR) RT-PCR was performed using the same template without adding reverse transcriptase.

Cloning of approximately 100 bp upstream sequence encompassing each of the three upstream motifs 1–3 in front of chimeric ncRNA::GFP reporter genes suggested that all three motifs have independent transcriptional activity. To test whether the PSE/UM1 is sufficient for ncRNA expression, we made a reporter construct consisting of a fragment of the CeN7 locus including 90 bp upstream and 67 bp transcribed (i.e. -90 to +67 bp; Additional file 1) sequence. The CeN7 locus encodes an SL2 RNA with a TATA-less PSE/UM1 upstream sequence. The fragment was inserted into plasmid pPD95_77, which contains a GFP ORF, thereby creating a CeN7::GFP chimeric reporter gene (henceforth CeN7_100). The recombined gene was co-injected with the rol-6 marker gene into young adult hermaphrodite gonads, and transgenic strains were selected based on the presence of the roller phenotype. Reporter gene expression was examined by reverse transcription polymerase chain reaction (RT-PCR) with one primer located in the CeN7 fragment and the other primer in the plasmid sequence, using RNA extracted from transgenic *C. elegans *strains (Figure 2). To confirm that the observed transcriptional activation was not a spurious result, we further selected an additional PSE/UM1 SL2 RNA locus (CeN16-1). The construct CeN16-1_100 was made as above from a fragment containing 119 bp upstream sequence and 60 bp transcribed sequence, and expression of the fusion reporter (CeN16-1::GFP) was confirmed with RT-PCR (Figure 2). Transgenic strains containing the empty plasmid pPD95_77 were also obtained the same way as described above, but no reporter gene expression was observed by RT-PCR.

The upstream motif 2 is composed of two sub-motifs with sequence and spacing similar to that of the A and B boxes of the tRNA promoter. To address the transcriptional activity of UM2, two constructs were made. One (CeN37_100) was made from a fragment containing 130 bp upstream sequence and 56 bp transcribed sequence from the snoRNA locus CeN37, the other (CeN55_100) from a fragment containing 134 bp upstream sequence and 89 bp transcribed sequence from the snoRNA locus CeN55. RT-PCR with one primer located in the CeN37 and CeN55 fragment and the other primer in the plasmid sequence demonstrated the expression of transgenes CeN37::GFP and CeN55::GFP, respectively (Figure 2).

Upstream motif 3 is exclusively found at stem-bulge RNA (sbRNA) loci. To determine the transcriptional activity of UM3, 140 bp and 120 bp of the upstream sequence of the sbRNA loci CeN74-2 and CeN72, respectively, was cloned in front of the GFP ORF in pPD95_77 to yield constructs CeN74-2_100 and CeN72_100 (the constructs also included 32 and 36 bp of the respective transcribed sbRNA sequences, fused to the GFP reporter gene). RT-PCR verified the expression of both reporter fusions (Figure 2).

### Additional regulatory elements

To assay whether the sequence upstream of each motif might contain additional regulatory elements, we also constructed plasmids containing approximately 300 to 1000 bp fragments of the 5'-flanking sequence from the CeN7, CeN37 and CeN74-2 loci, respectively. Transgenes expression levels were assayed by quantitative RT-PCR (qRT-PCR), and for each construct, 2 to 5 transgenic lines were tested.

**Figure 3:**
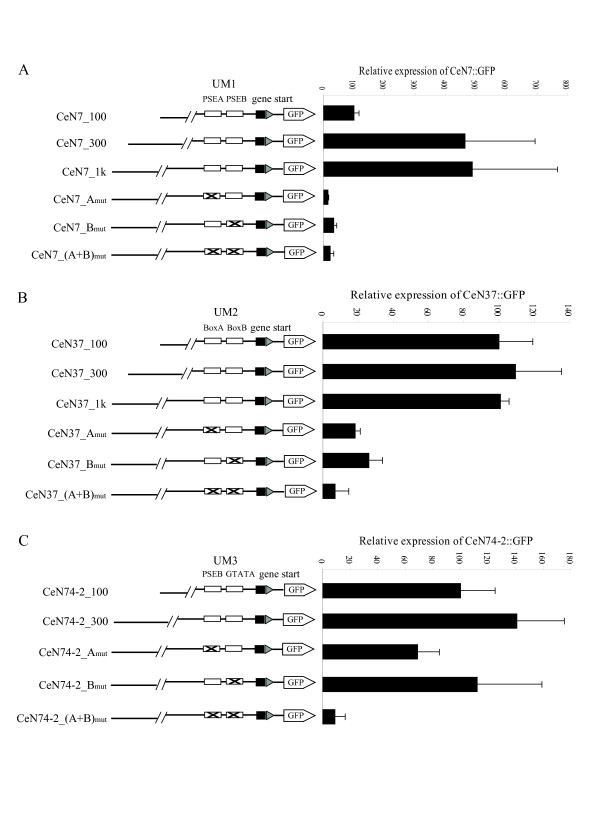
**Analysis of upstream motif promoter activity**. The panels show the effects of additional upstream sequence length, and of mutation of sub-motifs, on the transcriptional activity of each of the three promoters PSE/UM1, UM2 and UM3, respectively. (A) UM1 from the SL2 RNA locus CeN7. "PSEA" and "PSEB" are the most invariant sequence elements in the PSE/UM1. (B) UM2 from the snoRNA locus CeN37. "BoxA" and "BoxB" show the two sub-motifs in UM2. (C) UM3 from the sbRNA locus CeN74-2. "PSEB" and "GTATA" are the most invariant sequence elements of the UM3. "Gene start" corresponds to the first 69, 32 and 56 bp of the CeN7, CeN74-2 and CeN37 mature transcripts, respectively. Relative expression levels are calculated by normalizing the qRT-PCR intensities of each CeNX_100 construct to 100. Sub-motif mutations were performed on the constructs containing the longest upstream sequences (e.g. CeN7_1k) mainly by converting each purine and pyrimidine residue to the opposite purine and pyrimidine, respectively (i.e. A to G, and vice versa).

In the case of UM1, qRT-PCR of RNA from transgenic strains showed that the expression driven by CeN7_300 (containing a 264 bp 5' flanking fragment) was 4–5 fold higher than expression driven by CeN7_100 (Figure 3A), strongly suggesting the existence of an enhancer located within the 90~264 bp upstream region. However, a search for 5' end features in this region of PSE/UM1 ncRNAs in *C. elegans *failed to yield any common motifs (see Methods for details). The expression driven by CeN7_1k showed an almost identical level to that of CeN7_300, indicating that no additional regulatory elements exist within the 264~1000 bp upstream of the ncRNA locus. In the case of UM3, no significant increase in expression was found by increasing the length of the upstream fragment to 245 bp (CeN74-2_300; Figure 3B). Similarly, qRT-PCR indicated no increase in expression of the UM2 locus when the upstream region was extended to 294 bp (CeN37_300) and 989 bp (CeN37_1k; Figure 3C). These observations suggest that for the two assumed RNA polymerase III-driving promoters (i.e. UM2 and UM3) most or all the promoter activity resides within 100 bp of the 5'end of the respective annotated loci.

### Mutational analysis of promoter sub-motifs

Each of the three upstream motifs contains two sub-motifs whose base pairs are particularly invariant among loci. To investigate the contribution of the PSEA and PSEB sub-motifs to the overall PSE/UM1 transcriptional activity, we mutated each of the two sub-motifs mainly by converting each purine and pyrimidine residue to its opposite purine and pyrimidine, respectively (i.e. A to G, and *vice versa*), in the context of CeN7_1k. The corresponding constructs were labeled as CeN7_A_mut_, CeN7_B_mut _and CeN7_(A+B)_mut_. Mutation of either of the two sub-motifs (PSEA and PSEB) reduced expression to 3~7 % of that of CeN7_1k, suggesting that both PSEA and PSEB are required for the transcription of PSE/UM1 loci (Figure 3A).

The sub-motifs in UM2 promoter strongly resemble the A and B box motifs of the tRNA internal promoter. Mutation of either of these two motifs in the context of CeN37_1k caused a strongl reduction in the expression levels of the mutant constructs (18 % for CeN37_A_mut _and 26 % for CeN37_B_mut _compared with CeN37_1k; Figure 3B). Concomitant mutation of both sub-motifs (CeN37_(A+B)_mut_) reduced the expression level to 7 % compared with CeN37_1k.

Of the two sub-motifs in UM3, one strongly resembles the PSEB of the PSE/UM1, whereas the second sub-motif has the consensus sequence GTATA. We mutated both motifs in the same fashion as described above, and observed the effect on expression in the context of CeN74-2_300. Abolishing the PSEB-like sub-motif (CeN74-2_A_mut_) reduced the expression level to 49 % compared with that of non-mutated CeN74-2_300. Mutation of the GTATA sub-motif produced an even more modest reduction (79 % compared to that of non-mutated CeN74-2_300) than PSEB. Mutating both sub-motifs simultaneously, however, resulted in near abolishment of expression of the reporter (CeN74-2_(A+B)_mut_; Figure 3C). The results suggest a certain redundancy in regulatory activity between the two sub-motifs of UM3, but also that at least one of the sub-motifs must be present for expression of the downstream locus to occur at an appreciable rate.

### Transcription start sites of the upstream motif loci

**Figure 4:**
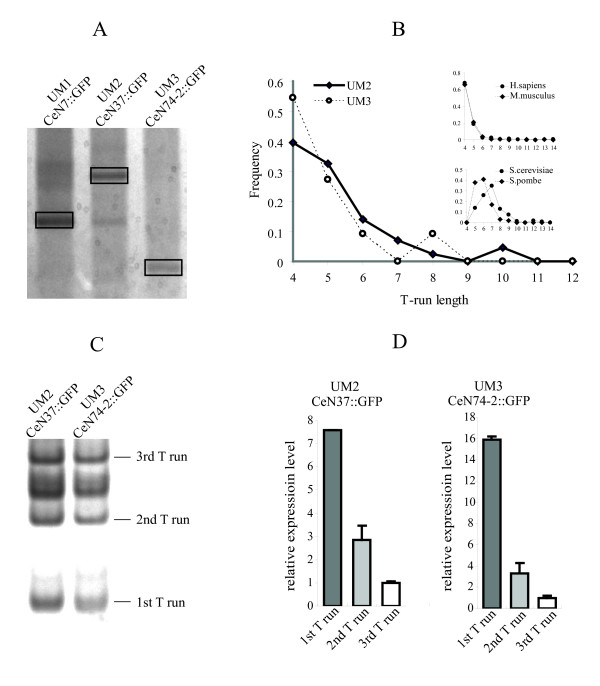
**UM1-3 transcript start and terminator analysis.** (A) 5'RACE analysis of the UM1-3 reporter gene transcripts (CeN7::GFP, CeN37::GFP and CeN74-2::GFP). Boxed bands correspond to the 5'RACE products of each reporter. The transcription start sites indicated by the 5'RACE bands of reporter constructs CeN7::GFP (UM1) and CeN74-2::GFP (UM3) are identical to the 5'ends of the mature endogenous CeN7 and CeN74-2 trancripts, respectively. The 5'RACE band from the CeN37::GFP (UM2) reporter constructed corresponds to a transcript starting 70 bp upstream of the 5'end of the mature endogenous CeN37 transcript (The lower band in the same lane is an unspecific band). (B) Distribution of *C. elegans *UM2 and UM3 ncRNAs terminator lengths. Frequency distribution analysis of poly(dT) terminator length was conducted on UM2 and UM3 ncRNA loci possessing a single RNA polymerase III termination signal starting within the first 40 bp downstream of the genomic sequence corresponding to the mature ncRNA. Inserted figures show data based on the analysis in ref [14]. (C) 3' RACE of CeN74-2_300 (UM3) and CeN37_1k (UM2). Bands corresponding to transcripts terminated at the 1^st^, 2^nd ^and 3^rd ^"TTTT" run are indicated. (Sequence analysis suggests that the band between the second and third T run corresponds to transcript terminated at a T rich tract "TTCTTGTT"). (D) Relative expression level of transcripts terminated at the 1^st^, 2^nd ^and 3^rd ^T run. qRT-PCR was performed by reverse transcription using the primer located in front of the 1^st^, 2^nd ^and 3^rd ^T run, respectively, followed by a nested PCR.(see Methods for details).

5' RACE performed on non-mutated constructs found that the TSS of CeN7::GFP and CeN74-2::GFP were identical to 5' ends of the endogenous RNAs. For CeN37::GFP, however, the TSS was apparently located 70 bp upstream of the 5' end of mature endogenous RNA and 9 bp upstream of UM2 sequence itself (Figure 4A).

### UM2 – a possible remnant of tRNA-snoRNA dicistronic loci

**Figure 5:**
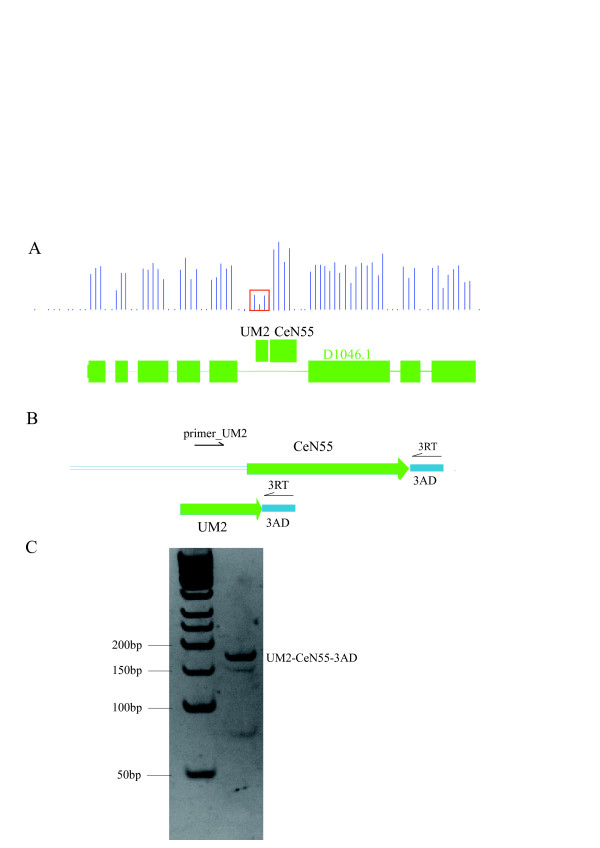
**The UM2-snoRNA transcript structure. **(A) The H/ACA snoRNA locus CeN55 is located in an intron of the protein coding gene D1046.1. Tiling array probe signal intensities indicate that the UM2 sequence upstream of snoRNA CeN55 might also be expressed (red box). (B) Model of RACE amplification of the putative UM2-snoRNA dicistronic fragments. 3AD is a 3'end adaptor ligated to the ncRNAs; 3RT is the primer complementary to 3AD, primer_UM2 located in the region of UM2. The RACE analysis was performed by reverse transcription using the 3RT as primer followed by PCR with primers primer_UM2 and 3RT. (C) Gel analysis of the RACE fragments.

As shown above, 5' RACE of the reporter transcript of a UM2 construct (CeN37_1k) found that transcription was initiated 70 bp upstream of the 5'end of the mature endogenous RNA, and even 9 bp upstream of the UM2 sequence. In plants, dicistronic tRNA-snoRNA transcripts, which are subsequently cleaved to yield mature tRNA and snoRNA have been described [32]. To investigate whether the UM2 is also internally located with regards to the primary transcript of the endogenous RNAs, we visually inspected the tiling microarray data [1] for possible evidence of transcription of the UM2 site itself. Although we found no indication of expression of the UM2 sequence at the CeN37 locus, we found other 21 instances with indications of some level of expression, 16 at known snoRNA loci and five preceding unannotated TUFs. In addition to CeN37, we therefore selected five candidate loci (CeN50-2, CeN39, CeN53, CeN55 and CeN119) for analysis by 3' RACE as indicated in Figure 5. If the dicistronic tRNA-snoRNA model also applies to the UM2-snoRNA transcripts, two RACE bands should be expected, one corresponding to the UM2-snoRNA primary transcript, the other to the UM2 fragment remaining after cleavage. Four (CeN39, CeN53, CeN55 and CeN119) of the 6 loci yielded RACE fragments of length expected if transcription initiation occurred at the start of, or upstream of the UM2 sequence, and sequencing confirmed a joint UM2-snoRNA transcript in all four cases. A band corresponding to a smaller fragment was observed in three cases, but none of the sequences included the UM2 fragment. Thus, the part of the primary transcript that contains the UM2 sequence is either rapidly degraded after cleavage, or the UM2-containing fraction of the primary transcript is removed by 5'exonuclease digestion during maturation of the snoRNA. 5' RACE analysis showed that the transcription of the UM2 loci starts 9~13 bp upstream of the first base in the box A-like sub-motif.

### Transcriptional termination

Transcripts derived from UM2 and UM3 loci are usually terminated at a run of several consecutive thymidine (T) residues, which is a property of loci transcribed by RNA polymerase III. The minimal number of consecutive T residues sufficient for termination of RNA polymerase III transcription varies among organisms, but analysis of known UM2 and UM3 loci suggests 4 consecutive Ts are sufficient for termination in *C. elegans *(Figure 4B). This is similar to what has been found in human and mouse, but is less than the 5–7 normally needed for termination in the yeast species (Figure 4B) [14]. In the plasmid pPD95_77 there are runs of four (or more) Ts located at variable distances from the plasmid multicloning site (MCS). To determine the actual termination sites of the UM2 and UM3 constructs, we performed 3'RACE on the resulting reporter transcripts. For CeN37_1k (UM2) the reporter transcript terminated at the first, second and third "TTTT" tracts (located 122, 177 and 236 nt downstream of TSS; Figure 4C). For the CeN74-2_300 (UM3) construct, reporter transcript termination was identical to what has been observed in CeN37_1k (Figure 4C). qRT-PCR of fragments corresponding to the different termination sites suggested that most of the UM2 and UM3 reporter transcripts terminated at the first "TTTT" tract (Figure 4D).

### PSE/UM1 drive expression of GFP

3'RACE of the UM1 reporter transcript gave no specific result, but RT-PCR indicated that at least a fraction of the GFP ORF was included in the transcript. We further investigated the expression of GFP in worms from 2–3 independent transgenic strains from each of the different constructs. No GFP expression was observed after genetic transformation with any of the UM2 (CeN37) and UM3 (CeN74-2) constructs. However, marked GFP expression was observed under fluorescent microscope for the CeN7_1k and CeN7_300 transgenic worms, and even the CeN7_100 transgenic worms showed weak GFP expression. To eliminate the possibility that the observed GFP expression was an effect of this particular PSE/UM1 locus, we tested another PSE/UM1 ncRNA locus, CeN16-1, whose upstream sequence was also able to drive GFP expression. 5'-end RACE performed on RNA extracted from CeN7_1k, CeN7_300 and CeN16-1_1k transgene worms showed that the transcription start site of the reporters were identical to that of the wild type ncRNA loci, suggesting that the transcriptional activity resulting in the observed GFP expression was the same as that driving the transcription of the endogenous ncRNAs.

**Figure 6:**
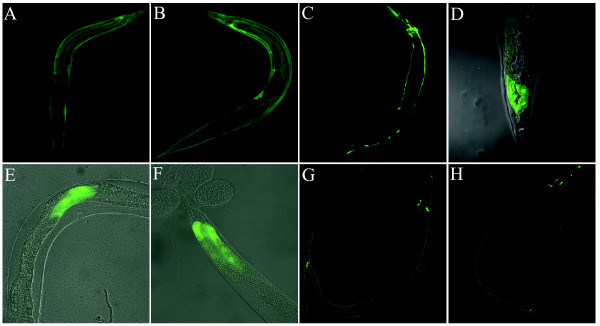
**In vivo expression patterns of different SL2 loci.** Expression of GFP under the UM1 promoters from the loci of (A) CeN7::GFP (CeN7_300), (B) CeN7::GFP (CeN7_1k), (C) CeN16-1::GFP (CeN16-1_1k), (D) CeN6::GFP (CeN6_1k), (E) CeN19::GFP (CeN19_1k), (F) CeN11::GFP (CeN11_1k) are shown. (G) GFP expression of CeN7_A_mut_, (H) GFP expression of CeN7_B _mut_.

Since PSE/UM1 could drive expression of GFP, we used UM1::GFP fusions to analyse the temporal-spatial expression pattern of related ncRNA loci having this upstream motif. One group of such loci are the *C. elegans *SL2 RNAs, which are a nematode-specific group of ncRNAs that function in trans-splicing of operonic mRNAs. There are around 20 SL2 RNA loci in *C. elegans *with slightly variable sequence characteristics. As far as is known, all participate in the same function, i.e. joining of an additional "exon" to the 5'end of internal (or non-first) mRNAs in operonic loci, but nothing appears to be known about the background for the numerous and variable SL2 RNA loci in the *C. elegans *genome. Previous experiments have demonstrated that different SL2 RNA genes show different temporal expression patterns [6,34,35,37], but little is known about their spatial expression patterns. We therefore examined the temporal-spatial expression of five SL2 RNA genes using promoter::GFP fusion constructs. The tested ncRNAs showed considerable variation in expression both in time and space. CeN7 is principally expressed in hypodermal cells, which is in agreement with previous *in situ *hybridization result [35], but also showed expression in skin muscles, excretory cells, head and ventral neuron cells (Figure 6A &6B; Additional file 2). The CeN16-1 locus was also active in excretory cells, and showed strong expression in the pharynx (Figure 6C; Additional file 2). Two other loci, CeN6 and CeN11, showed marked expression in intestinal muscles near the tail (Figure 6D &6F; Additional file 2), however, their temporal expression pattern differed, CeN11 showing visible expression from larval stage 3, while CeN6 showed no expression until the mature adult stage. The fifth SL2 RNA locus, CeN19, is expressed in intestinal muscles near the vulva (Figure 6E; Additional file 2). The temporal expression pattern is generally identical with previously northern and microarray analysis [6,34,35,37]. CeN7, CeN16-1 and CeN19 showed strong expression from an early stage of embryo development (Additional file 2) through the mature adult stage, whereas CeN6 and CeN11 both showed stage specific expression.

An interesting question is whether the transcriptional activity of the various SL2 RNA loci correlates with their target loci (i.e., the mRNAs to which the SL2 exons are spliced). To this end we downloaded spliced leader [38] and expressional data [19,39]. However, mRNA spliced to a specific SL2 RNA do not show any well correlated spatial expression patterns (Additional file 3), and even though it is possible to find SL2 RNA loci whose expression resembles one or a few of SL2 RNA [34] and mRNA temporal expression data [20,40,41] did not show any significant correlation between specific SL2 RNAs and their target mRNAs (Additional file 3).

We also observed that mutations in the PSEA and PSEB sub-motifs greatly changed the expression patterns of the CeN7 locus constructs (i.e. CeN7_A_mut_, CeN7_B_mut _and CeN7_(A+B)_mut_) compared with that of non-mutated constructs (i.e. CeN7_1k and CeN7_300). The CeN7_1k and CeN7_300 constructs were clearly expressed in hypodermal cells, skin muscles, excretory cells, head and ventral neurons (Figure 6A &6B; Additional file 2), however, the expression driven by CeN7 mutations was restricted to amphid and tail neuron cells (Figure 6G &6H). This suggests that the sequence characteristics of PSEA and PSEB are not only important for the general expression level of a locus, but may also influence where and when a locus is expressed.

## Discussion

Non-protein-coding RNAs are gaining in importance as functional elements in eukaryote cellular and organismal development [42-46]. *C. elegans *is one of the most important biological model systems for genetic and developmental studies, and it has recently been demonstrated that around 50 % of the transcriptional output in this organism cannot be identified as arising from protein-coding genes [34]. Among this vast amount of transcripts there are approximately 1400 relatively well-defined short non-coding transcripts. A notable fraction of these have strongly invariant upstream sequence motifs and this study has demonstrated that three of these motifs are able to activate *in vivo *transcription of otherwise inactive reporter genes.

The PSE/UM1 sequence is found at about 10 % of the 1400 known or putative noncoding loci, and is the most common promoter structure of *C. elegans *noncoding RNAs. Most of the PSE/UM1 ncRNAs are highly expressed, indicating this motif has relatively strong promoter activity. However, the expression levels of the PSE/UM1 loci vary greatly, even within the same functional class. For example, within the SL2 RNAs the expression levels of different loci can differ more than 10 fold [34]. Our analysis indicated that sequence elements within -90 to -264 bp relative to the transcription start site are also important for expression from the UM1-type promoter. At human snRNA loci a distal sequence element (DSE) is commonly found around 200 bp upstream of transcription start site [47,48], but a search in the region upstream of the PSE/UM1 in *C. elegans *failed to identify any common sequence motif (see Methods for details).

The PSE/UM1 promoter was also shown to drive expression of the protein (GFP) encoded in the reporter construct, suggesting that this motif activates RNA polymerase II expression. This agrees with previous evidence that the *C. elegans *PSE can drive expression of a *lac-Z *mRNA [35], but is the first demonstration in *C. elegans *of an active protein expressed under an ncRNA promoter. The transcriptional activity of two other upstream motifs (UM2 and UM3) was clearly demonstrated by RT-PCR, but no GFP expression was observed from these two promoters. The UM2 motif clearly resembles the tRNA promoter which is known to activate RNA polymerase III transcription through binding to the transcription factor IIIC [14]. Although polymerase specificity was not interrogated in this study, accumulated evidence [6] suggests that both UM2 and UM3 activate RNA polymerase III transcription. This is further corroborated by the finding that both endogenous loci and reporter constructs activated by these two promoters terminate transcription at runs of four (or more) consecutive T residues.

The observation that the PSE/UM1 promoter is able to drive GFP expression could allow for detailed analysis of the spatial-temporal expression of PSE/UM1 loci. Much genome wide data on the temporal expression of coding and noncoding genes in *C. elegans *have been obtained through Serial Analysis of Gene Expression (SAGE) and microarrays [3,6,20,34,40,41,49-51], and large scale expression profiling aimed at the spatial expression pattern of protein coding genes have been performed by several groups [19,22,23,52-54]. However, there are almost no data available on the spatial expression patterns of ncRNAs. As demonstrated in this study, a GFP expression under PSE/UM1 promoters from SL2 RNA agreed well with reported *in situ *hybridization data [35], and was able to specify the detailed expression characteristics of several SL2 RNA loci, in some cases down to the cellular level. Determination of the spatial and temporal expression patterns of ncRNAs can be the key to their function, and this assay could be a very convenient tool for *in vivo *analysis of the expression pattern of the TATA-less UM1 loci. An additional aspect is that there appear to be few reports on embryo-specific promoters in *C. elegans*, and the finding that some of the PSE/UM1 promoters are active at this stage may be of practical use to other research within the field.

As a promoter the UM2 sequence represents a particularly interesting case. The sub-motifs of the UM2 resemble the tRNA internal promoter elements box A and B, and 5'RACE analysis showed that the UM2 sequence was embedded in the primary reporter transcript. Detailed re-analysis of the *C. elegans *tiling microarray data [34] indicated the existence of similar primary transcripts arising from several genomic UM2 loci, and subsequent 3'RACE verified this. The fact that a UM2-containing primary transcript could not be identified at all inspected loci (including the endogenous CeN37 locus used for the reporter construct) could owe to the primary transcripts being inherently unstable and rapidly degraded during snoRNA maturation [33]. The *C. elegans *UM2 primary transcripts resemble the dicstronic tRNA-snoRNA transcripts found at a few plant and yeast loci [29,32,55,56]. Recently, several *Drosophila *snoRNAs were found to derive from longer RNA polymerase III transcripts, some of which were shown to contain an element similar to the B box of the tRNA promoter [57], and closer inspection of the same sequences in *Drosophila *also suggested the presence of an A box-like element upstream of the B box-like sequence at several loci. In the yeast genome there has also been reported one snoRNA whose RNA polymerase III transcription is driven by an A+B box configuration [13,55]. *In vitro *experiments in yeast demonstrated that box A alone can direct efficient TFIIIC-dependent transcription, while box B is dispensable [13], however, *in vivo *experiments found that both box A and B are required for the downstream transcripts accumulation [13]. The *in vivo *mutational analysis reported here also suggests a requirement for both box A and B for efficient transcription of UM2 loci in *C. elegans*. Given that similar snoRNA promoter characteristics are found in animals, plants and fungi could point to a very old promoter strategy that utilised a tRNA-like promoter to drive expression of snoRNA genes.

Although the promoter activities of ncRNAs, in particular those of snRNAs, have been analysed in great detail in a variety of organisms such as human, Drosophila and yeast for a couple of decades, little work has been done in *C. elegans*. Our work demonstrated that all the three investigated upstream motifs in *C. elegans *are transcriptionally active. However, this work has only concentrated on promoters with distinctive sequence characteristics, and the great majority of intergenic ncRNA loci show no obvious upstream motifs. What sequence elements are important and which protein complexes are recruited to initiate the transcription of such loci is still not known, and further efforts are needed for better a understanding of the *C. elegans *ncRNA transcriptional mechanism.

## Conclusion

We demonstrate here the transcriptional activities of three putative ncRNA promoters. Mutational analysis found that the most invariant sub-motifs of the UM1 and UM2 sequences are required for the downstream genes transcription, while the two sub-motifs of UM3 show redundancy with respect to transcriptional activity. We also show that UM1 can drive expression of GFP, suggesting that this promoter drive RNA polymerase II transcription, and the UM1::GFP fusions have shown to be useful in determine the temporal-spatial expression patterns of UM1 ncRNAs. UM2 and UM3 can not drive the expression of GFP, and termination at "TTTT" tract strongly suggests RNA polymerase III transcription. Several cases of tRNA-snoRNA dicistronic transcription pattern have been found, and it is likely that most of the UM2 snoRNAs apply this model of transcription.

## Methods

### Worm strains

Standard nematode cultivating conditions were used as described in ref [58]. The strains used were N2 and UNC-76.

### Construction of plasmids

Individual PCR reactions were performed in 50 or 100 ul reaction volume using wild type *C. elegans *(N2 strain) genomic DNA as template. PCR products corresponding to the desired fragments were digested with enzyme HindIII and BamHI, and then cloned into the promoter-less vector pPD95_77 (kindly provided by Andrew Fire) upstream of the GFP reporter gene. The constructs were verified by sequencing. Mutations were performed using standard PCR procedures. The primers used in this work are listed in Additional file 4.

### Transgenic *C. elegans *lines

Reporter constructs were injected at 50 ng/ul together with 50 ng/ul transformation marker pRF4 [rol-6(su1006)] or unc-76. Stable lines of transgenic worms were established as described previously [59]. For each construct, 2~5 transgenic lines were analysed.

### RT-PCR/qRT-PCR

RNA was extracted from transgenic worms according to Trizol (Invitrogen) protocol. RNA digested with DNase I (Fermentas) was used as template for RT-PCR (Qiagen one step RT-PCR kit). For qRT-PCR, reverse transcription was performed using primers 95_77_2_R and U2_R, and the cDNA was used as template for the qPCR according to qPCR mix protocol (Qiagen QuantiTect SYBR Green PCR Kit). When the expression levels of transcripts of different length were to be compared, a nested approach using internal primer yielding identical length PCR products were used for the qPCR step (following reverse transcription). The reactions were carried out on an MJ Research Opticon TM 2.

### 3'-RACE

3'-RACE was performed as described [1] with minor modifications. Briefly, DNase I digested total RNA was ligated to the 3' end adaptor 3AD, and the ligated RNA was reverse transcribed into complementary DNA (cDNA) with a primer (3RT) complementary to the 3' adaptor 3AD. First round PCRs were performed by using primers 3RT and pPD95_77_1_F. Nested PCR was then performed, using 3RT and pPD95_77_2_F as primers, and the first round PCR products as template. The PCR products were analysed on a PAGE gel, and candidate bands were recovered and sequenced.

### 5'-RACE

DNase I digested total RNA was reverse transcribed into cDNA with primers pPD95_77_2_R, pPD95_77_3_R and pPD95_77_4_R, respectively. The cDNA was treated with terminal DNA transferase (Fermentas) to add a 3'end poly(A) tail. First round PCR was performed with 3'CDS primer and the corresponding primer used for reverse transcription. Second round PCR was then carried out using the diluted first round PCR products as template, and 3'CDS and pPD95_77_1_R as primers. The second round PCR products were analysed, cloned and sequenced as above.

### Computational analysis

*C. elegans *genome annotation and sequence data were downloaded from Wormbase (version WS140) [39]. The MEME motif discovery tool (version 3.0.13) [25] was used to search for conserved motifs within and upstream of the ncRNA loci. The search for common motifs acting as enhancer on 54 UM1/PSE ncRNAs was carried out on 100~300 bp upstream sequences of these loci.

## Authors' contributions

TL, HH, YW and HZ performed the experiments. TL and HH interpreted the results. TL, HH and GS drafted the manuscript. RC and GS directed the design of the study. All authors have read and approved the final manuscript.

## Supplementary Material

Additional file 1 Genomic location. The data provided describes the genomic locations of all ncRNA loci used in this work. Click here for file

Additional file 2 In vivo expression patterns. The data provided shows the expression patterns of multiple lines of each SL2 RNAs. Click here for file

Additional file 3 trans-spliced genes and related SL2 RNAs. The data provided shows the expression patterns of trans-spliced genes and related SL2 RNAs. Click here for file

Additional file 4 Primers. The data provided shows the primers used in this work. Click here for file
