# Supplementary material for: In vivo analysis of Caenorhabditis elegans noncoding RNA promoter motifs
Source: BMC Mol Biol. 2008 Aug 5;9:71. doi: 10.1186/1471-2199-9-71 (PMC2527325; doi:10.1186/1471-2199-9-71)
Supplement: Additional file 1 — Genomic location. The data provided describes the genomic locations of all ncRNA loci used in this work. [file 1471-2199-9-71-S1.pdf]

Genomic locations of all ncRNA loci used in this work.

UM	Construct	chr	start	end
UM1	CeN7_100	III	12250250	12250406
	CeN7_300	III	12250075	12250406
	CeN7_1k	III	12249340	12250406
	CeN16-1_100	II	5543449	5543627
	CeN16-1_1k	II	5542295	5543627
	CeN6_1k	I	9066948	9066034
	CeN11_1k	IV	5318419	5317305
	CeN19_1k	I	13304632	13306059
UM2	CeN37_100	IV	8375656	8375471
	CeN37_300	IV	8375820	8375471
	CeN37_1k	IV	8376515	8375471
	CeN55_100	IV	8927113	8927335
UM3	CeN74-2_100	X	14477324	14477495
	CeN74-2_300	X	14477219	14477495
	CeN72_100	V	5590554	5590709
