# Supplementary material for: In vivo analysis of Caenorhabditis elegans noncoding RNA promoter motifs
Source: BMC Mol Biol. 2008 Aug 5;9:71. doi: 10.1186/1471-2199-9-71 (PMC2527325; doi:10.1186/1471-2199-9-71)
Supplement: Additional file 2 — In vivo expression patterns. The data provided shows the expression patterns of multiple lines of each SL2 RNAs. [file 1471-2199-9-71-S2.pdf]

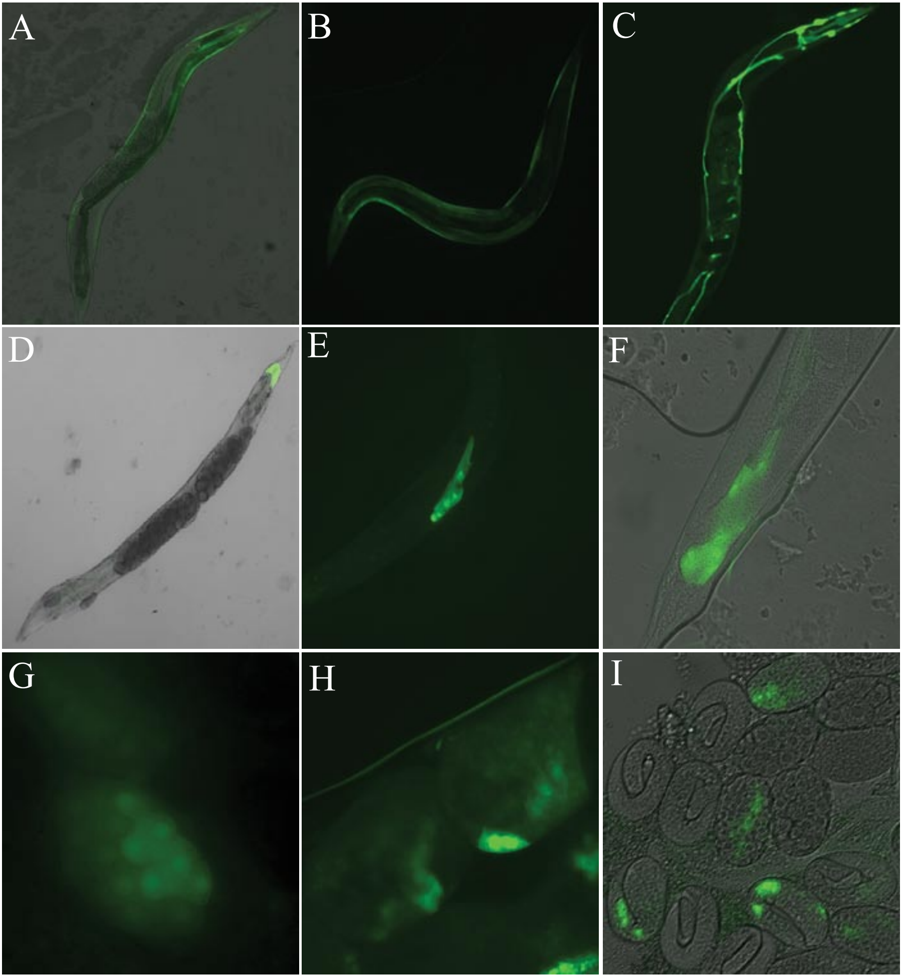

In vivo expression patterns of different SL2 loci (different lines from Figure 6 in the manuscript). Expression of GFP under the UM1 promoters from the loci of (A) CeN7::GFP (CeN7_300), (B) CeN7::GFP (CeN7_1k), (C) CeN16-1::GFP (CeN16-1_1k), (D) CeN6::GFP (CeN6_1k), (E) CeN19::GFP (CeN19_1k), (F) CeN11::GFP (CeN11_1k) are shown. Expression of (G) CeN7::GFP, (H) CeN19::GFP and (I) CeN16-1::GFP in embryos, respectively.
