# Supplementary material for: In vivo analysis of Caenorhabditis elegans noncoding RNA promoter motifs
Source: BMC Mol Biol. 2008 Aug 5;9:71. doi: 10.1186/1471-2199-9-71 (PMC2527325; doi:10.1186/1471-2199-9-71)
Supplement: Additional file 4 — Primers. The data provided shows the primers used in this work. [file 1471-2199-9-71-S4.pdf]

All primers used in this work.

**name of primers**	**sequence (from 5'-end to 3'-end)**
CeN7_1k	GCGAAGCTTTCCTCGATGCGAGTTCTTC
CeN7_300	GCGAAGCTTATAGAGAAAATTTGCGGC
CeN7_100	GCGAAGCTTGTCTCCCCCCACAAAACACA
CeN7_R	GGCGGATCCTTTTATTCGACACACTCT
CeN7_95_77	GGTTTTAACCCAGTTTAACCA
CeN37_1k	GCGAAGCTTACCGTAAGTCAGAAGATAGTT
CeN37_300	GCGAAGCTTAGTACGTGAGCGATTGAG
CeN37_100	GCGAAGCTTTGTACCCATCCCATTGAGAG
CeN37_R	GGCGGATCCCATCATTTCCCTTCTACA
CeN37_95_77	TCTATGTGTACTCGCTCGTCG
CeN74-2_300	GCGAAGCTTAAAGTTGGGCTGACACAT
CeN74-2_100	GCGAAGCTTAGTTGCAGAACGCTTATCTA
CeN74-2_R	GGCGGATCCAACTTGATAACCCACTGA
CeN74-2_95_77	TGTATCGGTCCGGCGTCA
pPD95_77_1_F	GATCCCCGGGATTGGCCA
pPD95_77_2_F	GCCAAAGGACCCAAAGGT
pPD95_77_1_R	CTTTGGGTCCTTTGGCCA
pPD95_77_2_R	GAAAATGTTCTATGTTATGTTAGTATC
pPD95_77_3_R	TCAAGGGTCCTCCTGAAAAT
pPD95_77_4_R	CACTGACAGAAAATTTGTGCCC
CeN7_Amut_F	ACAAAACACAGCACCCGATACGGTTGAGCAAATGTCCGTCCAC
CeN7_Amut_R	GTGGACGGACATTTGCTCAACCGTATCGGGTGCTGTGTTTTGT
CeN7_Bmut_F	CGGAACCCGGCAAACACTGATCCACAGCAGATTAGTGG
CeN7_Bmut_R	CCACTAATCTGCTGTGGATCAGTGTTTGCCGGGTTCCG
CeN7_Bmut_afterAmut_F	TACGGTTGAGCAAACACTGATCCACAGCAGATTAGTGG
CeN7_Bmut_afterAmut_R	CCACTAATCTGCTGTGGATCAGTGTTTGCTCAACCGTA
CeN37_Amut_F	TCTGCTGTTGCACGCTCAACACGACAATTAAGAGACGTGGTCTTCA
CeN37_Amut_R	TGAAGACCACGTCTCTTAATTGTCGTGTTGAGCGTGCAACAGCAGA
CeN37_Bmut_F	GGTCTTCAGTAATACTACAAAGGTCGTATGGTTCTCTCACTGCATCTATGTG
CeN37_Bmut_R	CACATAGATGCAGTGAGAGAACCATACGACCTTTGTAGTATTACTGAAGACC
CeN74-2_Amut_F	GCCATTGCACGAAAAACACTCATTATCTCACTCTCCCTTGGTAT
CeN74-2_Amut_R	ATACCAAGGGAGAGTGAGATAATGAGTGTTTTTCGTGCAATGGC
CeN74-2_GTATAmut_F	GCCTCACTCTCCCTTGACTCCAGAACAGCCTACAATCACCG
CeN74-2_GTATAmut_R	CGGTGATTGTAGGCTGTTCTGGAGTCAAGGGAGAGTGAGGC
CeN74-2_GTATAmut_afterAmut_F	ATCTCACTCTCCCTTGACTCCAGAACAGCCTACAATCACCG
CeN74-2_GTATAmut_afterAmut_R	CGGTGATTGTAGGCTGTTCTGGAGTCAAGGGAGAGTGAGAT
CeN16-1_100	GGCAAGCTTGTTACTCAAGGTACGCTGGAGTT
CeN16-1_1k	GGCAAGCTTCCTGAACTCGTTTTGGTTAC
CeN16-1_R	GCGGGATCCTTGACACTCTCTTTCGAA
CeN16-1_95_77	GGTTTTAACCCAGTTACTCA
CeN55_100	GGCAAGCTTTTAGTGCTGTGACTCTCTGC
CeN55_R	GGCGGATCCATATTGCACCTGCTACCA

CeN55_95_77	GCCTAGAAACTCAGCGGTGT
CeN72_100	GGCAAGCTTATGGGTTATTATCCTGTG
CeN72_R	GCGGGATCCCACAGGATAATAACCCAT
CeN72_95_77	ATCATCGGTCCGGTGTTG
CeN6_1k	GCCCTGCAGGTTAAACTCTGATAAGGACGCC
CeN6_R	GCGGGATCCCGAAGAAGCTGTTCCAA
CeN11_1k	GGCAAGCTTTCATAACGATTCAGTTGCTGC
CeN11_R	GCGGGATCCCAATCTTTCGAATGGTC
CeN19_1k	GGCAAGCTTGCATTGTTTTCCTCTAGGAG
CeN19_R	GCGGGATCCAGAGAGTGTCGATTGTG
3RT	CCCTGTGAGCTCGTGGTCAA
3AD	UUUUGACCACGAGCTCACAGGG
3'CDS	GGCCACGCGTCGACTAGTAC(T)17V (V=A,C,G)
CeN18(U2)_F	ATCGCTTCTTCGGCTTAT
CeN18(U2)_R	GTCTTCCCTAGGTTCCAAA
CeN119_UM2_F	ATGTGTGCGCTGTGTGCA
CeN39_UM2_F	CCGTTAGTCGCTGGTTCGT
CeN50-2_UM2_F	GGCACCCACTGGTTCAATT
CeN53_UM2_F	CCGGTCAGTGTGTTTGCTGT
CeN55_UM2_F	AGTCAGCAGTCCGCGTGAT
CeN37_UM2_F	GAGACGTGGTCTTCAGTAATACT
CeN119_UM2_R	ACATGCTGCCGGCCTCGAA
CeN55_UM2_R	ATCACGCGGACTGCTGACT
CeN53_UM2_R	CCAGAGGGATTGAACTCTCAAC
CeN50-2_UM2_R	ACACAGGGAATTGAACCAGTGG
CeN39_UM2_R	ATCCGCCATAAACTCTACAGC
CeN37_UM2_R	AGTATTACTGAAGACCACGTCTC
